# Prevalence of pre-eclampsia and adverse pregnancy outcomes in women with pre-existing cardiomyopathy: a multi-centre retrospective cohort study

**DOI:** 10.1038/s41598-022-26606-z

**Published:** 2023-01-04

**Authors:** Laura Ormesher, Sarah Vause, Suzanne Higson, Anna Roberts, Bernard Clarke, Stephanie Curtis, Victoria Ordonez, Faiza Ansari, Thomas R. Everett, Claire Hordern, Lucy Mackillop, Victoria Stern, Tessa Bonnett, Alice Reid, Suzanne Wallace, Ebruba Oyekan, Hannah Douglas, Matthew Cauldwell, Maya Reddy, Kirsten Palmer, Maggie Simpson, Janet Brennand, Laura Minns, Leisa Freeman, Sarah Murray, Nirmala Mary, James Castleman, Katie R. Morris, Elizabeth Haslett, Christopher Cassidy, Edward D. Johnstone, Jenny E. Myers

**Affiliations:** 1 Maternal & Fetal Health Research Centre, Division of Developmental Biology and Medicine, University of Manchester, Manchester, UK; 2 Saint Mary’s Hospital, Manchester University NHS Foundation Trust, Manchester, UK; 3 Manchester Heart Centre, Manchester University NHS Foundation Trust, Manchester, UK; 4 Division of Cardiovascular Sciences, University of Manchester, Manchester, UK; 5 Bristol Heart Institute, Bristol Royal Infirmary, Bristol, UK; 6 Leeds Teaching Hospitals NHS Trust, Leeds, UK; 7 Oxford University Hospitals NHS Foundation Trust, Oxford, UK; 8 Academic Unit of Developmental and Reproductive Medicine, University of Sheffield, Sheffield, UK; 9 Department of Obstetrics, Nottingham University Hospitals NHS Trust, Nottingham, UK; 10 Guy’s and St Thomas’ NHS Foundation Trust, London, UK; 11 St George’s University Hospitals NHS Foundation Trust, London, UK; 12 Monash Women’s, Monash Health, Monash University, Melbourne, Australia; 13 Scottish Adult Congenital Cardiac Service, Golden Jubilee National Hospital, Glasgow, UK; 14 Queen Elizabeth University Hospital, NHS Greater Glasgow & Clyde, Glasgow, UK; 15 Department of Cardiology, Norfolk& Norwich University Hospital Foundation Trust, Norwich, UK; 16 Royal Infirmary of Edinburgh, NHS Lothian University Hospitals Division, Edinburgh, UK; 17 Birmingham Women’s and Children’s Hospital NHS Foundation Trust, Birmingham, UK; 18 Institute of Applied Health Research, University of Birmingham, Birmingham, UK; 19 Blackpool Teaching Hospitals NHS Foundation Trust, Blackpool, UK

**Keywords:** Cardiomyopathies, Pre-eclampsia, Heart failure, Echocardiography

## Abstract

Pre-eclampsia is associated with postnatal cardiac dysfunction; however, the nature of this relationship remains uncertain. This multicentre retrospective cohort study aimed to determine the prevalence of pre-eclampsia in women with pre-existing cardiac dysfunction (left ventricular ejection fraction < 55%) and explore the relationship between pregnancy outcome and pre-pregnancy cardiac phenotype. In this cohort of 282 pregnancies, pre-eclampsia prevalence was not significantly increased (4.6% [95% C.I 2.2–7.0%] vs. population prevalence of 4.6% [95% C.I. 2.7–8.2], *p* = 0.99); 12/13 women had concurrent obstetric/medical risk factors for pre-eclampsia. The prevalence of preterm pre-eclampsia (< 37 weeks) and fetal growth restriction (FGR) was increased (1.8% vs. 0.7%, *p* = 0.03; 15.2% vs. 5.5%, *p* < 0.001, respectively). Neither systolic nor diastolic function correlated with pregnancy outcome. Antenatal ß blockers (n = 116) were associated with lower birthweight Z score (adjusted difference − 0.31 [95% C.I. − 0.61 to − 0.01], *p* = 0.04). To conclude, this study demonstrated a modest increase in preterm pre-eclampsia and significant increase in FGR in women with pre-existing cardiac dysfunction. Our results do not necessarily support a causal relationship between cardiac dysfunction and pre-eclampsia, especially given the population’s background risk status. The mechanism underpinning the relationship between cardiac dysfunction and FGR merits further research but could be influenced by concomitant ß blocker use.

## Introduction

There is abundant observational data linking pre-eclampsia with postnatal maternal cardiac dysfunction^1–3^ and long-term cardiovascular risk^4–15^. However, the mechanistic link between cardiac dysfunction and pre-eclampsia remains inconclusive: it is unclear whether it is causal^16,17^ or consequential^18,19^. There is evidence to support a pre-eclampsia dose–effect: the more severe pre-eclampsia phenotypes (determined by presence of severe features^5,8,11–13,20^, gestation at onset^4,6,7,10^, reduced fetal size^13,21^ and recurrence^8,22^ are associated with increased future cardiovascular risk. Indeed, preterm pre-eclampsia (< 37 weeks’ gestation) is associated with worse maternal diastolic dysfunction^3,23^ and potentially worse cardiac remodelling^24^, although evidence for the latter is more conflicted^3,25^.

Animal studies have also sought to investigate the direction of causation, with mixed results^26–30^. An alternative approach is to examine pre-eclampsia rates in women with pre-existing cardiac dysfunction. Pre-eclampsia and fetal growth restriction (FGR) are presumed to be placental in origin^31–34^. In this way, if inadequate cardiac function contributes significantly to impaired placental development, women with pre-existing cardiac dysfunction should have disproportionately increased pre-eclampsia and FGR rates. A number of large retrospective registry studies^35–43^ have previously investigated obstetric outcomes in women with known cardiac disease. However, pre-eclampsia prevalence was not the primary focus of these studies, and therefore to our knowledge, no one has correlated pre-pregnancy cardiac parameters with pregnancy outcome and pre-eclampsia risk factors have largely been overlooked.

The aim of this study was to determine the prevalence of pre-eclampsia and FGR (clinical proxies for placental dysfunction) in women with pre-existing cardiac dysfunction, aiming to improve:

1. Our understanding of the relationship between cardiac and placental function, to inform future preventative and therapeutic strategies;
2. Counselling of women with cardiac disease and their families, before and during pregnancy.

## Results

**Figure 1:**
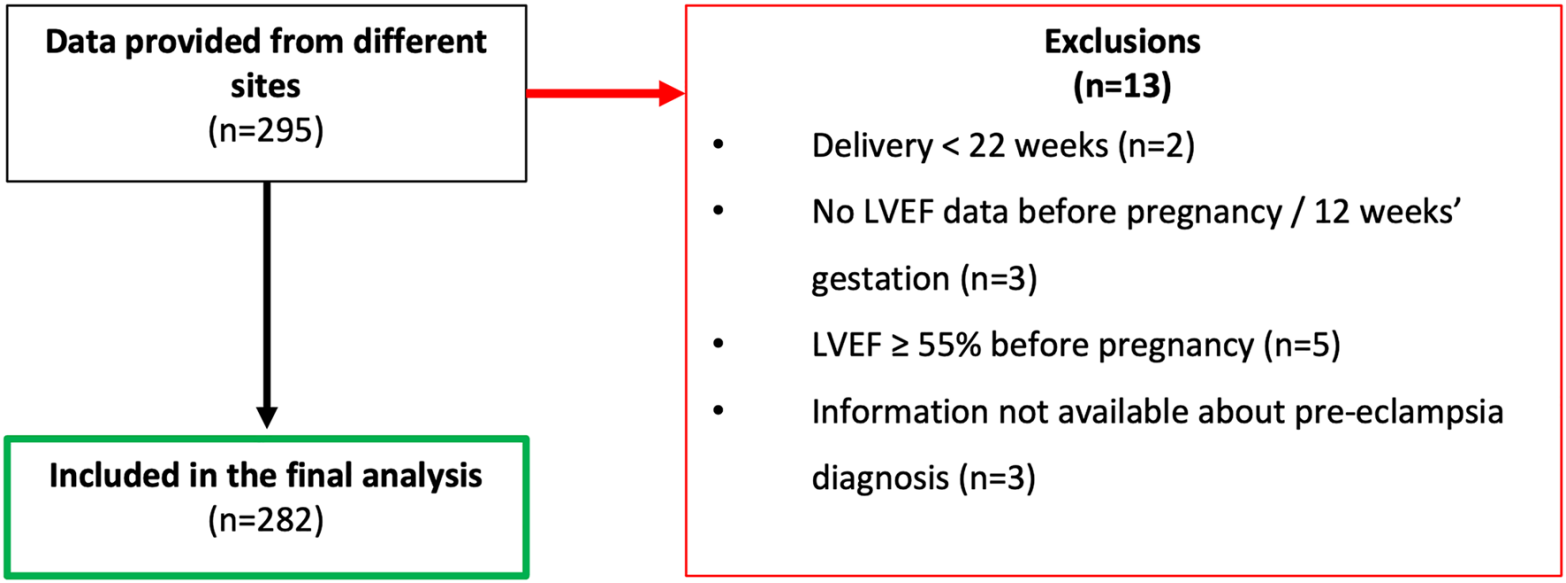
Consort diagram.

The study cohort included 282 pregnancies from 244 women (Fig. 1). Supplementary Table S1 describes the spread of participants from different sites. Results were derived from the 282 pregnancies but were not altered by only including each woman’s first pregnancy.

### Demographics and baseline characteristics

Distribution of baseline characteristics of the cohort are summarised in Table 1. Left ventricular ejection fraction (LVEF) data were derived from early pregnancy (< 12 weeks) in 16/282 (5.7%) women, where pre-pregnancy echocardiography data were not available. DCM affected 156/282 (55.3%) of the cohort. Of those with dilated cardiomyopathy (DCM), 50/156 (32.1%) were familial, 3/156 (1.9%) were idiopathic, 63/156 (40.4%) were acquired and 35/156 (22.4%) were due to previous PPCM.

**Table 1:** Baseline characteristics.

	DCM (n = 156)		All (n = 282)
**Demographics**			
Age at delivery (years)	30.3 ± 6.4	30.1 ± 6.2	
Ethnicity	White	115/156 (73.7%)	222/282 (78.8%)
	Black	14/156 (9.0%)	18/282 (6.4%)
	Asian	8/156 (5.1%)	15/282 (5.3%)
	Other	6/156 (3.8%)	14/282 (5.0%)
	Unknown	13/156 (8.3%)	13/282 (4.6%)
Booking BMI (kg/m^2^)*		25.8 (17.0–48.7)	25.8 (17.0–50.8)
Smoker during pregnancy		17/147 (11.6%)	53/270 (19.6%)
**Cardiac diagnosis**			
Dilated cardiomyopathy		–	156/282 (55.3%)
Congenital		–	36/282 (12.8%)
Ischaemic		–	12/282 (4.3%)
Hypertensive		–	3/282 (1.1%)
Valvular		–	32/282 (11.3%)
Genetic without DCM		–	21/282 (7.4%)
Other acquired without DCM		–	22/282 (7.8%)
**Medical history**			
Chronic hypertension		12/153 (7.8%)	20/279 (7.2%)
Pre-existing renal disease		9/153 (5.9%)	15/277 (5.4%)
Pre-existing proteinuria		9/150 (6.0%)	13/269 (4.8%)
Pre-existing diabetes		5/155 (3.2%)	8/281 (2.8%)
Autoimmune disease		8/153 (5.2%)	9/276 (3.3%)
Booking sBP (mmHg)		110.0 ± 14.1	109.7 ± 13.6
Booking dBP (mmHg)		67.3 ± 10.1	68.3 ± 10.0
**Obstetric history**			
Nulliparous		38/156 (24.4%)	83/282 (29.1%)
High risk for pre-eclampsia		32/153 (20.9%)	74/279 (26.5%)
At least one moderate risk factor for pre-eclampsia		78/120 (65.0%)	158/250 (63.2%)
Previous pre-eclampsia (if multiparous)		12/116 (10.3%)	24/191 (12.6%)
Previous SGA < 10th centile (if multiparous)		19/89 (21.4%)	41/158 (25.9%)
Previous FGR < 3rd centile (if multiparous)		12/88 (13.6%)	25/157 (15.9%)

Frequencies: n/N (%). Mean ± standard deviation.

*Median (range). Denominators vary between variables due to missing data. Congenital heart disease encompassed structural defects including coarctation of the aorta, patent ductus arteriosus, ventricular septal defect, pulmonary atresia, tetralogy of Fallot, transposition of the great arteries and truncus arteriosus. Genetic causes without DCM (dilated cardiomyopathy) include: hypertrophic obstructive cardiomyopathy, arrhythmogenic cardiomyopathy and left ventricular non-compaction cardiomyopathy. Other acquired causes without DCM include: previous PPCM, drug-induced and inflammatory.

^†^High risk for pre-eclampsia is defined by presence of: pre-existing hypertension, renal, vascular or autoimmune disease, diabetes, previous pre-eclampsia, or two moderate risk factors.

^‡^Moderate risk factors include: nulliparity, age ≥ 40 years, multi-fetal pregnancy, BMI ≥ 35 kg/m^2^.

*BMI* Body mass index, *DCM* Dilated cardiomyopathy, *PPCM* Peripartum cardiomyopathy, *sBP* Systolic blood pressure, *dBP* Diastolic blood pressure, *mmHg* Millimetres of mercury, *SGA* Small for gestational age, *FGR* Fetal growth restriction.

The majority of women were New York Heart Association (NYHA) functional classification I (109/282 [38.7%]); 64/282 (22.7%) were class II; 14/282 (5.0%) were class III, and 95/282 (33.7%) were unknown. Table 2 summarises the baseline echocardiography parameters of the cohort. Thirty-two (11.3%) women had severe systolic dysfunction (LVEF ≤ 35%); 110/282 (39.0%) had impaired LVEF and 140/282 (49.6%) had borderline LVEF. Echocardiography data, beyond LVEF was not available for every participant. Concentric remodelling/hypertrophy affected 13/126 (10.3%) and eccentric hypertrophy affected 51/126 (40.5%) women.

**Table 2:** Echocardiography measures of cardiac structure and function prior to pregnancy or before 12 weeks’ gestation.

Echocardiography parameters		DCM (n = 156)		All (n = 282)	
		Mean ± SD/median (range)/N (%)	N	Mean ± SD/median (range)/N (%)	N
Cardiac remodelling	Concentric remodelling	2 (2.5%)	79	8 (6.3%)	126
	Concentric hypertrophy	3 (3.8%)	79	6 (4.8%)	126
	Eccentric hypertrophy	36 (45.6%)	79	51 (40.5%)	126
LVIDd (cm)		5.58 ± 0.57	95	5.24 ± 0.70	190
LVIDs (cm)		4.31 ± 0.68	83	3.89 ± 0.84	160
PWd* (cm)		0.90 (0.40–1.30)	84	0.80 (0.40–1.60)	163
IVSd* (cm)		0.80 (0.40–1.40)	89	0.82 (0.40–2.50)	167
LVM* (g)		181.40 (72.34–286.04)	83	159.60 (52.09–376.33)	156
LVMi* (g/m^2^)		94.83 (39.14–182.38)	79	90.90 (34.40–182.38)	126
RWT*		0.31 (0.13–0.59)	84	0.31 (0.13–0.64)	163
E/A*		1.50 (0.61–7.00)	55	1.50 (0.61–7.00)	96
E/E'*		8.00 (3.00–16.00)	31	8.00 (3.0–24.30)	51
Left atrial dilatation		12 (7.8%)	39	16 (24.2%)	66
Aortic stenosis		–	–	16 (6.9%)	232
Aortic regurgitation		–	–	39 (16.8%)	232
Mitral stenosis		–	–	9 (3.8%)	234
Mitral regurgitation		–	–	48 (20.4%)	235
Pulmonary stenosis		–	–	4 (1.7%)	233
Pulmonary regurgitation		–	–	27 (17.9%)	151
Tricuspid stenosis		–	–	0 (0.0%)	232
Tricuspid regurgitation		–	–	62 (27.7%)	224
Cardiac output (L/minute)		5.53	1	4.47 ± 1.13	5
Stroke volume (mL)		75.4 ± 11.8	5	60.43 ± 15.64	16
TAPSE* (cm)		2.03 (0.70–20.0)	41	2.30 (0.70–23.00)	96

Frequencies: N (%). Mean ± standard deviation.

*Median (range). Mild, moderate and severe valvular abnormalities were included; trivial and physiological regurgitation/stenosis were excluded. Echocardiography data was not available for all women, therefore the number included in the analysis (N) is recorded for each parameter.

*SD* Standard deviation, *LVIDd* Left ventricular internal diameter in end-diastole, *LVIDs* Left ventricular internal diameter in end-systole, *PWd* Posterior wall thickness in end-diastole, *IVSd* Interventricular septal wall thickness in end-diastole, *LVM* Left ventricular mass, *LVMi* LVM indexed to body surface area, *RWT* Relative wall thickness, *E/A* Early to late diastolic filling ratio, *E/E’* Early diastolic filling to early diastolic mitral annular velocity ratio, *TAPSE* Tricuspid annular plane systolic excursion.

ß blockers were taken by 116/243 (47.7%) women antenatally. Information on the type of ß blocker was available for 77/116 (66.4%) women; bisoprolol was the most commonly prescribed ß blocker (68/77, 88.3%). Antenatal aspirin was taken in 102/257 (39.7%) pregnancies (Supplementary Table S2). All women with ischaemic heart disease (n = 12) took aspirin during pregnancy.

### Pregnancy outcomes

Table 3 summarises the pregnancy outcomes of the cohort. The median gestation at delivery was 38^22–42^ completed weeks and 123/273 (45.0%) delivered by Caesarean section. Thirteen (4.6%) women developed pre-eclampsia. Five (38.5%) of these women delivered before 37 weeks, 3/13 (23.1%) before 34 weeks and 12/13 (92.3%) had risk factors for pre-eclampsia (including hypertension, renal disease, antiphospholipid syndrome, obesity, nulliparity and diabetes; Supplementary Table S3). Within the cohort, 74/279 (26.5%) met the criteria for antenatal aspirin^44^. For those women where antenatal aspirin was indicated due to pre-eclampsia risk factors^44^ and for whom pre-eclampsia risk factors and aspirin use were known, 45/69 (65.2%) took aspirin antenatally. Pre-eclampsia prevalence observed in this cohort was not significantly increased compared with the general population^45^ (4.6% [95%C.I 2.2–7.0%] vs. 4.6% [95% C.I. 2.7–8.2], *p* = 0.99). On the other hand, preterm pre-eclampsia prevalence was increased relative to population prevalence^46^ (1.8% [95% C.I. 0.2–3.3] vs. 0.7% [95% C.I. 0.6–0.8], *p* = 0.03); this did not retain statistical significance when only women with DCM were included (Supplementary Table S4). Three (60.0%) of those with preterm pre-eclampsia had co-existent FGR. Prevalence of FGR and small for gestational age (SGA) in women with pre-existing cardiac impairment were higher than that of the background population (FGR: 15.2% [95% C.I. 10.9–19.5%] vs. 5.5% [95% C.I. 5.3–5.7], *p* < 0.001; SGA: 32.0% [95% C.I. 26.4–37.5%] vs. 18.2% [95% C.I. 17.9–18.6], *p* < 0.001).

**Table 3:** Pregnancy outcomes of the cohort.

Pregnancy outcome		DCM (n = 156)	All (n = 282)
Gestation at delivery* (completed weeks)		38 (27—42)	38 (22—42)
Delivery < 37 weeks		43/152 (28.3%)	73/278 (27.3%)
Delivery < 34 weeks		12/152 (7.9%)	30/278 (10.8%)
Iatrogenic delivery < 34 weeks		11/152 (7.2%)	21/276 (7.6%)
Female sex		49/107 (45.8%)	118/233 (50.6%)
Mode of delivery	Emergency C-section	21/147 (13.4%)	37/273 (13.6%)
	Elective C-section	44/147 (29.9%)	86/273 (31.5%)
	Operative vaginal delivery	11/147 (7.5%)	26/273 (9.5%)
	Breech vaginal delivery	0/147 (0.0%)	4/273 (1.5%)
	Spontaneous vaginal delivery	71/147 (48.3%)	120/273 (44.0%)
Indication for delivery	Spontaneous	67/156 (43.0%)	106/282 (37.6%)
	Routine	47/156 (30.1%)	104/282 (36.9%)
	Fetal concerns	5/156 (3.2%)	7/282 (2.5%)
	Worsening maternal cardiac disease	32/156 (20.5%)	38/282 (13.5%)
	Pre-eclampsia	4/156 (2.6%)	12/282 (4.3%)
	Other maternal disease	0/156 (0.0%)	2/282 (0.7%)
	Unknown	1/156 (0.6%)	13/282 (4.6%)
EBL*		400 (50–4000)	400 (40–4000)
Multiple pregnancy		0/156 (0.0%)	2/282 (0.7%)
**Perinatal outcomes**			
Birthweight centile*		25 (0–99)	24 (0–99)
Birthweight Z score		− 0.68 ± 1.18	− 0.71 ± 1.15
Birthweight centile < 10th		42/144 (29.2%)	86/269 (32.0%)
Birthweight centile < 3rd		21/144 (14.6%)	41/269 (15.2%)
NICU admission		28/106 (26.4%)	58/198 (29.3%)
Stillbirth		0/156 (0.0%)	0 (0.0%)
NND		1/156 (0.6%)	2/282 (0.7%)
**Maternal outcomes**			
Pre-eclampsia		5/156 (3.2%)	13/282 (4.6%)
Severe pre-eclampsia		4/156 (2.6%)	9/282 (3.2%)
Early-onset pre-eclampsia (delivery < 34 weeks)		2/156 (1.3%)	5/282 (1.8%)
Preterm pre-eclampsia (delivery < 37 weeks)		3/156 (1.9%)	5/282 (1.8%)
Eclampsia		1/156 (0.6%)	1/282 (0.4%)
Gestation at pre-eclampsia diagnosis* (weeks + days)		33 + 0 (31 + 0 − 35 + 5)	33 + 0 (31 + 0 − 36 + 5)
Gestational diabetes		7/118 (5.9%)	13/221 (5.9%)
Placental abruption		2/119 (1.7%)	4/187 (2.1%)
PPROM		5/118 (2.5%)	10/221 (4.5%)

Frequencies: n/N (%).

*Median (range). Denominators vary between variables due to missing data.

*EBL* Estimated blood loss, *NICU* Neonatal intensive care, *NND* Neonatal death, *HELLP* Haemolysis, elevated liver enzymes and low platelets; PPROM, premature rupture of membranes.

Of the 13 women with pre-eclampsia, six were nulliparous. There was no significant difference in pregnancy outcome between nulliparous and multiparous women in this cohort (pre-eclampsia: 7.2% vs. 3.5%, *p* = 0.18; preterm pre-eclampsia: 2.4% vs. 1.5%, *p* = 0.27; FGR: 19.8% vs. 13.3%, *p* = 0.18; preterm FGR: 11.3% vs. 5.7%, *p* = 0.11). Similarly, when only nulliparous women were included in the analysis (n = 83), pre-eclampsia prevalence remained comparable with background population (7.2% [95% C.I. 1.6–12.8%] vs. 4.6% [95% C.I. 2.7–8.2], *p* = 0.65). Pre-eclampsia and preterm pre-eclampsia rates in the nulliparous women were also comparable with the SCOPE study^47^, which included nulliparous low-risk women (pre-eclampsia: 7.2% [95% C.I. 1.6–12.8%] vs. 5.3% [95% C.I. 4.6–6.0%], *p* = 0.44; preterm pre-eclampsia: 2.4% [95% C.I. − 0.9–5.7%] vs. 1.3% [95% C.I. 1.0–1.7%], *p* = 0.40). Pre-eclampsia rates were also comparable with the SCOPE study^47^ when the whole cohort was included (pre-eclampsia: 4.6% [95% C.I. 2.2–7.1%] vs. 5.3% [95% C.I. 4.6–6.0%], *p* = 0.62; preterm pre-eclampsia: 1.8% [95% C.I. 0.2–3.3%] vs. 1.3% [95% C.I. 1.0–1.7%], *p* = 0.54).

**Figure 2:**
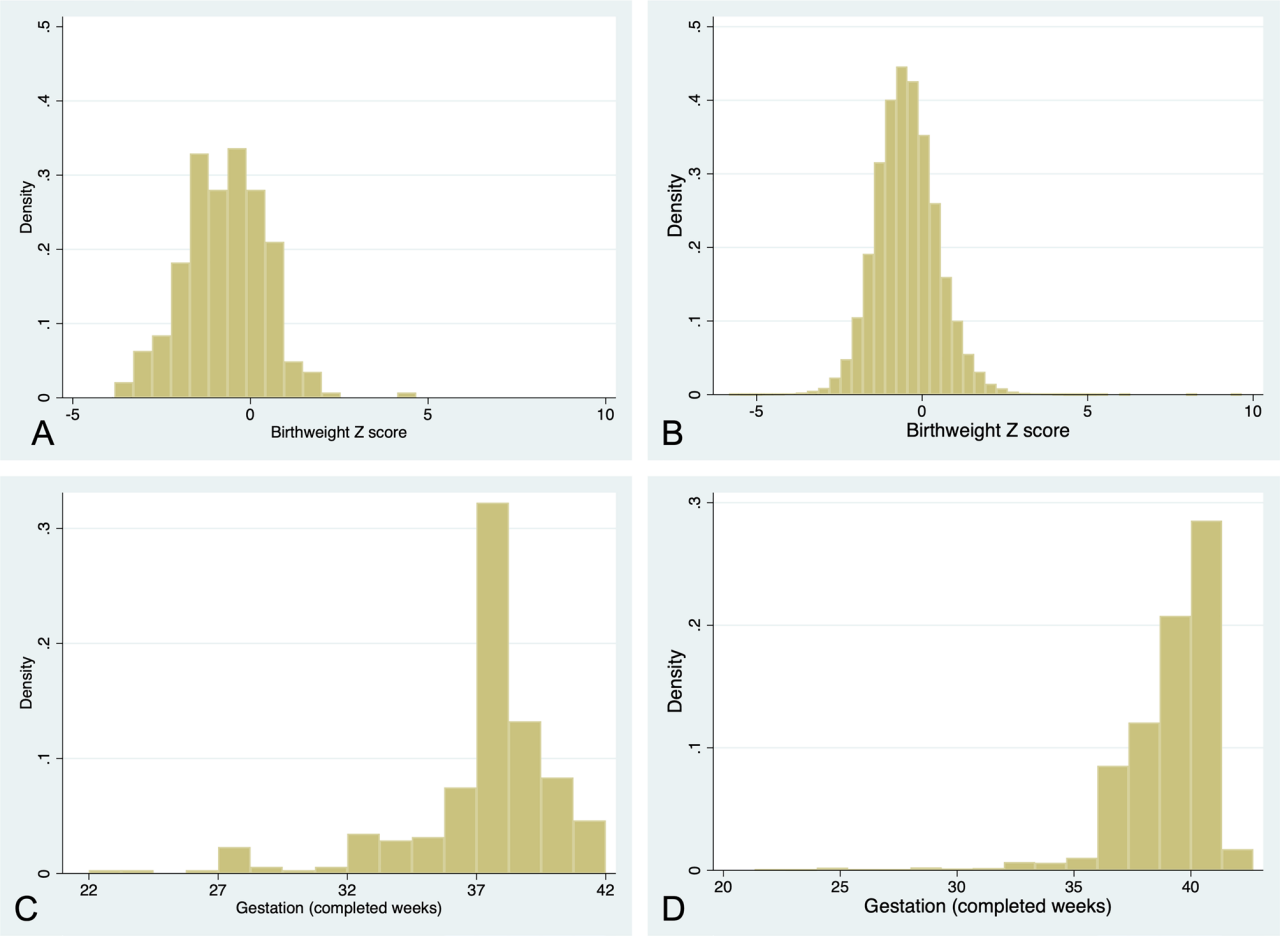
Histograms of (**A**) birthweight Z score in this cohort; (**B**) birthweight Z score in the background population; (**C**) gestation at delivery (completed weeks) in this cohort; and (**D**) gestation at delivery (completed weeks) in the background population. Background population distributions were derived from 5-year data (2016–2020) from St Mary’s Hospital, Manchester, UK.

Preterm delivery and preterm FGR were also more prevalent than in the background population (preterm delivery: 26.3% [95% C.I. 21.1–31.4%] vs. 8.2% [95% C.I. 8.0–8.5], *p* < 0.001; preterm FGR: 7.4% [95% C.I. 4.3–10.6%] vs. 1.5% [95% C.I. 1.4–0.2], *p* < 0.001). Pre-eclampsia affected 2/6 (33.3%) women with early-onset (< 34 weeks) FGR. Fifty-eight (21.3%) women had iatrogenic delivery < 37 weeks, of whom 45/58 (77.6%) were indicated by routine obstetric factors/maternal disease only. None of the women who suffered placental abruption (n = 4) had evidence of pre-eclampsia. Figure 2 illustrates the distribution of birthweight Z score and gestation at delivery in this cohort compared with the background population.

### Relationship between cardiac parameters and pregnancy outcome

Severity of left ventricular (LV) impairment did not correlate with prevalence of pre-eclampsia (Supplementary Table S5, *p* = 0.35), SGA (*p* = 0.24), FGR (*p* = 0.67), or preterm delivery < 34 weeks (*p* = 0.26). LV impairment severity also did not correlate with birthweight Z score or gestation at delivery. Ischaemic heart disease was not associated with pre-eclampsia prevalence but was associated with earlier gestation at delivery and lower birthweight Z score (log-transformed difference − 0.08 days [95% C.I. − 0.14 to − 0.03], *p* = 0.002 and difference -0.84 [95% C.I. − 1.50 to − 0.17], *p* = 0.01, respectively). The relationship between ischaemic heart disease and birthweight Z score did not persist after adjustment for smoking (adjusted difference -0.65 [95% C.I. − 1.34–0.03], *p* = 0.06). Neither DCM, valvular nor hypertensive cardiomyopathy correlated with pregnancy outcome (including pre-eclampsia diagnosis, birthweight Z score and gestation at delivery). NYHA status was not associated with prevalence of pre-eclampsia (*p* = 0.62) or FGR (*p* = 0.15).

In terms of pre-pregnancy echocardiography parameters, LV systolic and diastolic function did not correlate with pregnancy outcome (Table 4 and Supplementary Tables S6 and S7). Increased LV mass index (LVMi) weakly correlated with increased pre-eclampsia prevalence (5 g/m^2^ increase in LVMi: OR 1.18 [95% C.I. 1.01–1.38], *p* = 0.04; Table 4). This did not persist after adjustment for pre-eclampsia risk factors and booking mean arterial pressure (MAP; adjusted OR 1.16 [95% C.I. 0.98–1.37], *p* = 0.08). Aortic and mitral stenosis and pulmonary regurgitation were also associated with increased pre-eclampsia prevalence (OR 6.0 [95% C.I. 1.42–25.33], *p* = 0.02; OR 6.86 [95% C.I. 1.24–37.80], *p* = 0.02; and OR 4.00 [95% C.I. 1.10–14.57], respectively), but statistical significance was lost after adjustment as above (Supplementary Table S8). No pre-pregnancy echocardiography parameters correlated with birthweight Z score or gestation at delivery, except presence of concentric hypertrophy, which was associated with earlier gestation at delivery (log-transformed difference: -0.10 days [95% C.I. − 0.18 to − 0.03], *p* = 0.01) and tricuspid annular plane systolic excursion (TAPSE; a measure of right ventricular function) which weakly correlated with birthweight z score (coefficient: 0.04 [95% C.I. 0.00–0.08]).

**Table 4:** Relationship between echocardiography parameters and pre-eclampsia prevalence.

Echocardiography parameters	DCM			All		
	N	OR (95% C.I.)	*P* value	N	OR (95% C.I.)	*P* value
LVM (increment 10 g)	83	1.06 (0.86–1.31)	0.58	156	1.00 (0.87–1.14)	1.00
LVMi (increment 5 g/m^2^)	79	1.16 (0.98–1.38)	0.09	126	**1.18 (1.01–1.38)**	**0.04**
RWT (increment 0.1)	84	1.73 (–0.50–5.99)	0.39	163	0.89 (0.38–2.07)	0.79
E/A (increment 0.2)	55	1.04 (0.80–1.34)	0.79	96	1.06 (0.96–1.17)	0.27
E/E' (increment 1)	31	0.92 (0.60–1.40)	0.70	51	0.90 (0.66–1.23)	0.52
LV remodelling (compared to normal)	76			126		
Concentric hypertrophy (n = 6)		–	–		12.20 (0.66–225.73)	0.09
Concentric remodelling (n = 8)		**37.00 (1.22–1119.83)**	**0.04**		10.17 (0.56–184.01)	0.12
Eccentric hypertrophy (n = 49)		2.18 (0.19–25.10)	0.53		2.49 (0.22–28.27)	0.46
Left atrial enlargement*		–	–	54	**16.33 (0.81–330.35)**	**0.07**
Aortic stenosis† (n = 16)	–	–	–	232	**6.00 (1.42–25.33)**	**0.02**
Aortic regurgitation† (n = 37)	–	–	–	232	3.04 (0.84–10.93)	0.09
Mitral stenosis† (n = 7)	–	–	–	234	**6.86 (1.24–37.80)**	**0.03**
Mitral regurgitation† (n = 79)	–	–	–	229	2.26 (0.63–8.06)	0.21
Pulmonary stenosis† (n = 4)	–	–	–	233	7.30 (0.70–76.56)	0.10
Pulmonary regurgitation† (n = 27)	–	–	–	**227**	**4.00 (1.10–14.57)**	**0.04**
Tricuspid stenosis† (n = 0)	–	–	–	232	–	–
Tricuspid regurgitation† (n = 51)	–	–	–	224	3.36 (0.99–11.46)	0.05
TAPSE (increment 1 mm)	41	**0.74 (0.55–1.00)**	**0.048**	96	1.01 (1.00–1.02)	0.09

*Moderate left atrial enlargement compared with normal left atrial size.

^†^Compared to none/physiological valvular regurgitation/stenosis. N describes the number of observations included in the analysis. n describes the number of pregnancies affected by the condition. Bold text indicates statistical significance (*p* < 0.05).

*DCM* Dilated cardiomyopathy, *C.I.* Confidence interval, *LVM* Left ventricular mass, *LVMi* LVM indexed to body surface area, *RWT* Relative wall thickness, *E/A* Early to late diastolic filling ratio, *E/E’* Early diastolic filling to early diastolic mitral annular velocity ratio, *LA* Left atrium, *TAPSE* Tricuspid annular plane systolic excursion.

Pre-eclampsia prevalence was not associated with any antenatal medication. SGA and FGR were more prevalent in women taking ß blockers antenatally (SGA: 49/127 [38.6%] vs. 37/138 [26.8%], *p* = 0.04; FGR: 28/127 [22.0%] vs. 13/138 [9.4%], *p* = 0.005). Antenatal exposure to heparin was associated with a higher prevalence of FGR (16/65 [24.6%] vs. 22/196 [11.2%], *p* = 0.008). The relationship between heparin and birthweight Z score was lost after adjustment for pre-existing hypertension, underlying cardiac diagnosis and LV impairment severity (adjusted difference: − 0.29 [95% C.I. − 0.61–0.04], *p* = 0.09). Antenatal ß blocker use was associated with lower birthweight Z score, even after adjustment for pre-existing hypertension, underlying cardiac diagnosis and severity of LV impairment (adjusted difference − 0.31 [95% C.I. − 0.61 to − 0.01], *p* = 0.04). Furthermore, this relationship persisted after adjustment for smoking (adjusted difference − 0.38 [95% C.I. − 0.67 to − 0.09], *p* = 0.01) and when only those known to have taken bisoprolol were included (difference − 0.43 [95% C.I. − 0.76 to − 0.10], *p* = 0.01). Exploratory analyses comparing birthweight Z score in women who took bisoprolol antenatally with those who were known to take alternative ß blockers (n = 17) demonstrated no difference (difference 1.12 [95% C.I. − 0.42–2.68], *p* = 0.15). The association between antenatal ß blockers and earlier gestation at delivery was lost after adjustment for LVEF (adjusted log-transformed difference: − 0.02 [95% C.I. − 0.04–0.00], *p* = 0.09). Supplementary Table S9 compares maternal characteristics between those exposed to ß blockers antenatally and those not.

### Cardiac outcomes

Major adverse cardiovascular events (MACE) occurred in 3/282 (1.1%) pregnancies: one woman with LV non-compaction cardiomyopathy had a transient ischaemic attack and there were two maternal deaths (drug overdose and valvular thrombosis one month postpartum associated with a mechanical aortic valve). Thirty-six (12.8%) women developed acute heart failure and 14/282 (5.0%) developed pulmonary oedema. Sustained arrhythmia complicated 13/282 (4.6%) pregnancies. Table 5 summarises the prevalence of adverse cardiac outcomes according to severity and cause of cardiac dysfunction. Acute heart failure was most common in those with DCM (19.5%) and severely impaired LVEF (41.9%).

**Table 5:** Prevalence of adverse cardiac outcome depending on severity of LV impairment and cardiac diagnosis.

Severity of LV impairment	MACE	Acute heart failure	Pulmonary oedema	Sustained arrhythmia
Borderline	2/140 (1.4%)	**8/140 (5.7%)**	1/40 (2.5%)	10/140 (7.1%)
Impaired	0/110 (0.0%)	**15/109 (13.8%)**	0/108 (0.0%)	2/109 (1.8%)
Severely impaired	1/32 (3.1%)	**13/31 (41.9%)**	0/31 (0.0%)	2/31 (6.5%)
P value	0.27	** < 0.001**	0.61	0.15
**Primary cardiac diagnosis**				
DCM	**0/156 (0.0%)**	**30/154 (19.5%)**	1/153 (0.7%)	9/154 (5.8%)
Congenital	**0/36 (0.0%)**	**1/36 (2.8%)**	0/36 (0.0%)	3/36 (8.3%)
Ischaemic	**0/12 (0.0%)**	**1/12 (8.3%)**	0/12 (0.0%)	0/12 (0.0%)
Hypertensive	**0/3 (0.0%)**	**0/3 (0.0%)**	0/3 (0.0%)	0/3 (0.0%)
Valvular	**3/32 (9.4%)**	**3/32 (9.4%)**	0/32 (0.0%)	2/32 (6.3%)
Genetic without DCM	**0/21 (0.0%)**	**1/21 (4.8%)**	0/21 (0.0%)	0/21 (0.0%)
Other acquired without DCM	**0/22 (0.0%)**	**0/22 (0.0%)**	0/22 (0.0%)	0/22 (0.0%)
P value	**0.001**	**0.02**	0.99	0.65

Frequencies: n/N (%). Denominators vary between variables due to missing data. Severity of LVEF impairment was classified as: borderline (50–54%), impaired (36–49%) and severe (≤ 35%)^75^. Genetic causes without DCM (dilated cardiomyopathy) include: hypertrophic obstructive cardiomyopathy, arrhythmogenic cardiomyopathy and left ventricular non-compaction cardiomyopathy. Other acquired causes without DCM include: previous PPCM, drug-induced and inflammatory. *P* values represent comparison between LVEF impairment categories and cardiac diagnoses using Chi-square test. Bold text indicates statistical significance (*p* < 0.05).

*LVEF* Left ventricular ejection fraction, *SGA* Small for gestational age, *FGR* Fetal growth restriction; *DCM* Dilated cardiomyopathy.

## Discussion

This study describes a large retrospective cohort of pregnancies affected by pre-existing maternal heart disease. Pre-eclampsia prevalence was not increased compared to the general population, however preterm pre-eclampsia, SGA and FGR prevalence were. Routinely indicated preterm and early term delivery and Caesarean sections were common in this cohort. The severity of LV impairment did not correlate with any pregnancy outcome and there was only one case of pre-eclampsia amongst the pregnancies complicated by severely impaired baseline LVEF.

Antenatal ß blocker use was consistently associated with adverse pregnancy outcome, including increased SGA and FGR and reduced birthweight centile, despite adjustment for confounders. In those who were prescribed ß blockers, bisoprolol was the most commonly used agent.

Adverse cardiac events, although less frequent than previous reports in the literature^38,39^, were by no means uncommon, thereby endorsing close antenatal and postnatal surveillance in this high-risk group. Women with valvular disease were at particular increased risk of MACE and those with severely impaired LVEF had the highest risk of acute heart failure.

This was a relatively large multicentre study comprising data from 13 sites across the UK and Australia. Inclusion of 282 pregnancies affected by maternal cardiomyopathy allowed correlation of pre-existing cardiac parameters with pregnancy outcome. To our knowledge, this is the first study of women with pre-existing cardiac disease, in which the primary outcome is pre-eclampsia, thereby ensuring adjustment for pre-eclampsia risk factors, where appropriate. Although the retrospective nature of the study has its limitations, variables were pre-specified and confirmed by the clinical care team following careful review of clinical records. Heterogeneity of the cohort was compensated by subgroup analyses of women with DCM (thereby limiting confounding effects of structural heart disease on cardiac output (CO), LV geometry and function); these subgroup analyses demonstrated consistent findings with the whole cohort.

Unfortunately, background prevalence data for adverse pregnancy outcomes were not available from all sites; for this reason, the prevalence of SGA, FGR, preterm delivery and preterm FGR was estimated from five-year birth data from Saint Mary’s Hospital, Manchester. Although a tertiary centre with a high-risk population, Saint Mary’s Hospital was deemed an appropriate comparator as it contributed a large proportion of the cohort (99/282) and 11/13 of the sites are tertiary centres for Cardiology or Obstetrics. Pre-eclampsia is not reliably coded in UK hospital maternity information systems and therefore comparisons of rates of pre-eclampsia could only be made to published studies from comparable populations^45–47^. To compensate for this limitation, the influence and proportion of nulliparity and pre-eclampsia risk factors were explored.

If the link between cardiac dysfunction and pre-eclampsia is due to a problem with cardiovascular supply rather than demand, CO would be a useful pre-pregnancy parameter to determine this. Unfortunately, CO is not routinely reported in echocardiography and therefore it is not possible to explore the link between CO and pregnancy outcome, within this dataset. Additionally, the relatively mild LV functional impairment seen in this cohort (49.6% had LVEF 50–54%) may not have been enough to cause a significant drop in CO, thereby limiting any potential effect on uteroplacental perfusion. On the other hand, severity of LV impairment did not correlate with any measure of placental dysfunction, including pre-eclampsia, indicating a lack of causation.

Finally, for the purpose of this study, pre-eclampsia and FGR were considered clinical proxies for placental dysfunction, in the absence of confirmatory placental histology. This is due to the widely accepted theory of their mutual placental origin^31–34^, however this limits the ability to link pre-pregnancy cardiac parameters with distinct placental pathologies.

Pre-eclampsia prevalence was not increased in this cohort. However, routinely indicated early delivery in these women could have masked term pre-eclampsia. Additionally, frequent use of ß blockers could have masked late hypertension, thereby preventing a diagnosis of pre-eclampsia being made. On the other hand, the lack of association between systolic or diastolic function and pre-eclampsia, suggests that the cardiac dysfunction following pre-eclampsia^3,48,49^ is unlikely to be solely a consequence of pre-existing impairment. In contrast, increased LVMi was weakly associated with higher pre-eclampsia rates. This likely reflects pre-existing comorbidities, supported by the loss of relationship between LVMi and pre-eclampsia after adjustment for booking MAP and pre-eclampsia risk factors.

Although preterm pre-eclampsia rates were increased compared with those reported in the ASPRE trial^46^, this could in part be attributed to the increased prevalence of pre-eclampsia risk factors in this cohort (26.5% [95% C.I. 21.3–31.7%] vs. 4.0% [95% C.I. 3.8–4.2%], *p* < 0.001).

The high SGA and FGR rates in this cohort could be a consequence of reduced uteroplacental blood supply due to the underlying cardiomyopathy^50^ or concurrent medication (ß blockers)^51,52^, or contributed to by high smoking rates (20%). Alternatively, despite its ß1 cardio-selective nature^53^, bisoprolol could have a direct effect on the placental vasculature. This is supported by evidence of ß1 receptors^54,55^ in placental vasculature and placental vasoconstriction seen following exposure to ß blockers in vitro^56,57^. The potential negative effect of antenatal ß blocker use on fetal growth has long been considered^51,58–61^. A recent meta-analysis including 13 cohort studies demonstrated a significant increase in SGA associated with antenatal ß blocker use (OR 1.72 [95% C.I. 1.59–1.85], *p* < 0.001)^58^. It has been proposed that ß blocker subtypes are associated with varying risk^51,59^. Labetalol, which is an α and ß antagonist and partial ß2 agonist^57,62,63^, is commonly used as a first-line antihypertensive in pregnancy^44^. It is possible that the partial ß2 agonistic properties of labetalol induce vasodilation in placental and umbilical vasculature, thereby favourably increasing placental blood flow^57,64,65^. However atenolol, which selectively blocks ß1 adrenergic receptors, is not recommended in pregnancy^44^ due to negative associations with fetal growth^60,66,67^. The impact of bisoprolol, which was the most commonly prescribed ß blocker in this cohort, on fetal growth is less understood.

The rationale for ß blocker use in the context of cardiac dysfunction is to protect the heart against the deleterious effects of increased adrenergic activity, by reducing heart rate, blood pressure and myocardial oxygen demand^68^. It is therefore likely that continued antenatal use of ß blockers indicates a particular cardiac phenotype or degree of severity. However, the relationship between ß blockers and birthweight Z score persisted after adjustment for FGR risk factors and cardiac phenotype, indicating a direct mechanistic link between the two.

The relationship between antenatal heparin exposure and FGR was unexpected due to the wealth of existing data demonstrating no harmful effect of antenatal heparin on fetal growth^69–72^. Given the loss of significance after adjustment for confounders, this is unlikely to represent a causal relationship.

The lack of association between any measure of pre-pregnancy cardiac impairment and birthweight Z score/FGR makes a causal role of cardiac dysfunction in the development of FGR unlikely. Furthermore, if preterm FGR in this cohort shared the presumed aetiology of preterm pre-eclampsia, in which early placentation is affected by defective spiral artery remodelling^31^, the prevalence of co-existing hypertension (i.e. pre-eclampsia) should be higher. A third of women with early-onset FGR developed pre-eclampsia in this cohort, compared with 52–60% women in the early-onset FGR cohorts in TRUFFLE^73^ and STRIDER^74^. The lower-than-expected rate of pre-eclampsia in women with FGR suggests that late placental failure, rather than early placentation defects, may be a more significant cause of FGR in this cohort. This would also be supported by the association with ß blockers affecting third trimester growth^51^. In order to explore this further, measures of early placentation (including uterine artery Doppler, pregnancy-associated plasma protein-A, placental growth factor and placental pathology) need to be investigated in future cohorts.

In conclusion, this study provides valuable information to aid clinicians with pre-conception and antenatal counselling for women with cardiomyopathy. These women can be reassured that their risk of pre-eclampsia does not appear to be significantly increased, however serial ultrasound scanning is likely warranted to monitor for FGR. Preconception counselling should include information on the increased risk of acute heart failure in pregnancy for those with pre-existing cardiomyopathy (in particular severely impaired LVEF). The mechanism linking FGR and cardiac dysfunction remains unknown, however it could be attributed to reduced uteroplacental perfusion as a consequence of the underlying cardiac disease or concomitant ß blocker use. Further study is required to explore the effect of bisoprolol on the placenta. Finally, the absence of dose–effect demonstrated by lack of correlation between severity of cardiac dysfunction and pregnancy outcome does not support a causal role of cardiovascular dysfunction in the development of pre-eclampsia. Further study is needed to explore the mechanistic link between cardiac dysfunction and FGR.

## Methods

This was a multicentre retrospective cohort study, including 12 UK sites and one Australian site. Research was limited to use of previously collected, non-identifiable information. For this reason, it was approved by the UK Health Research Authority (HRA; IRAS ID 261380) and the Australian Human Research Ethics Committees (HREC/60940/MonH-2020-203642) without the need for UK Research Ethical Committee review (as per the UK Integrated Research Application System [IRAS]).

The need for informed consent was waived by the HRA and Australian Human Research Ethics Committee, as identifiable patient data were not accessed outside of the primary clinical care team. All methods were carried out in accordance with the "Caldicott Principles", the Data Protection Act and the General Data Protection Regulation. Patients were not involved in the conduct of this study.

### Study population

**Figure 3:**
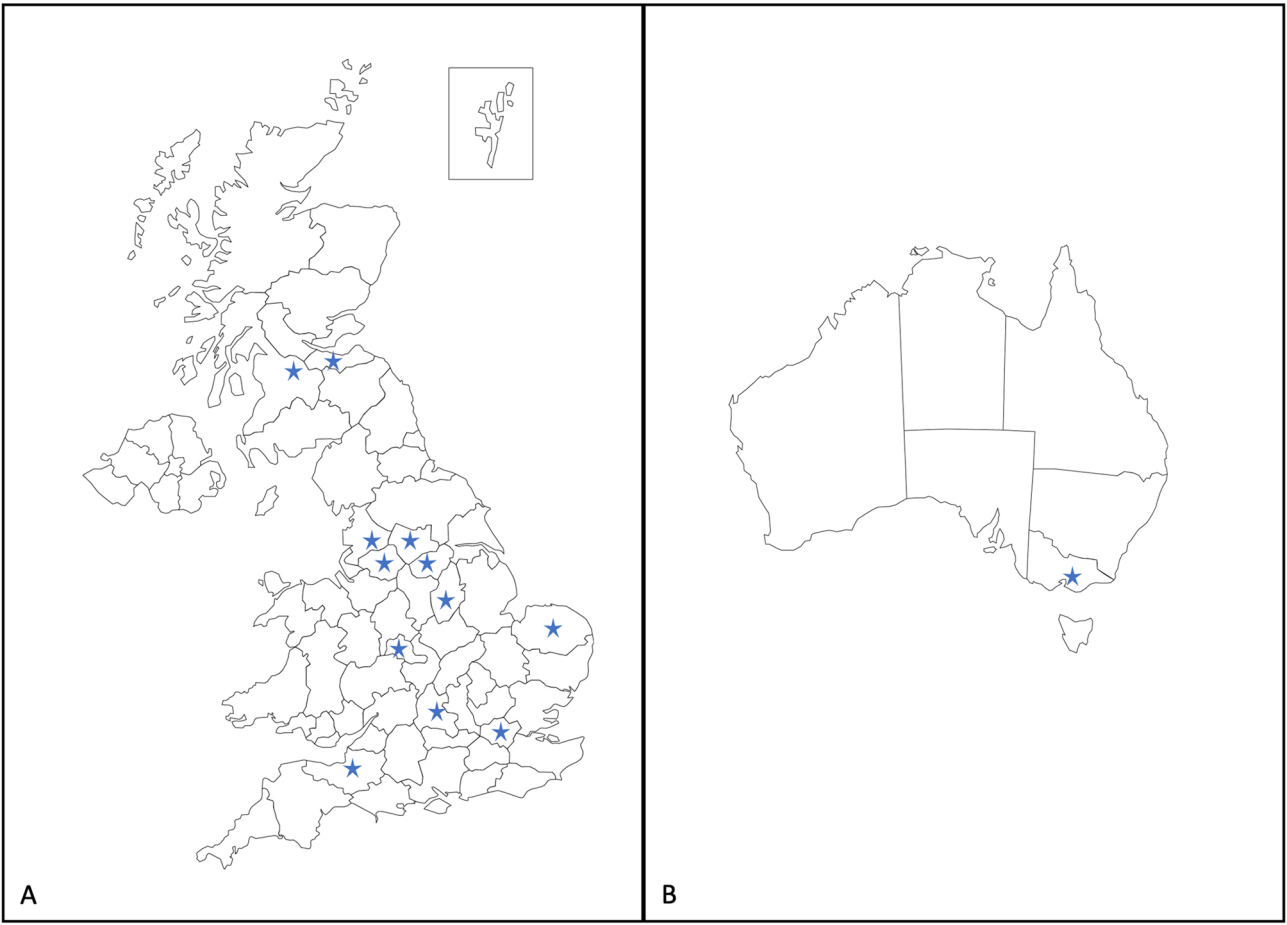
Map of participating sites in (**A**) UK and (**B**) Australia. Blue stars highlight the 13 hospitals that collected data for the study. Maps are modified from Bruce Jones Design^85^.

Women aged ≥ 16 years with pre-existing LV systolic impairment (LVEF < 55%), who had a pregnancy between January 2008 and December 2020, were included in the study. Women were excluded if they delivered before 22 weeks’ gestation or insufficient data were available. Data were collated from 13 sites in England, Scotland and Australia (Fig. 3).

Eligible participants were identified using different methods across sites, including via cardiac obstetric databases, clinic lists and ICD-10 codes (including heart failure and cardiomyopathy). Eligibility was checked following review of echocardiography reports, online clinical reports, clinic letters and case notes. Each site was given identical excel spreadsheets with pre-determined data fields to complete (Supplementary Table S10). Minimum data criteria included presence/absence of pre-eclampsia and evidence of LVEF < 55% pre-pregnancy or < 12 weeks’ gestation.

### Cardiac classifications

LV impairment was categorised, as per British Society of Echocardiography (BSE) guidelines^75^, as borderline (50–54%), impaired (36–49%) and severely impaired (≤ 35%). DCM was defined as a combination of dilated left ventricle (LV internal diameter in end-diastole [LVIDd] > 5.2 cm)^76^ and systolic dysfunction, or evidence of DCM diagnosis by a cardiologist in the case notes. DCM was further categorised into familial, idiopathic, acquired (secondary to infection, chemotherapy, alcohol or iron overload) and previous peripartum cardiomyopathy (PPCM). As per the American Heart Association (AHA)^77^ and BSE guidance^78^, congenital, ischaemic, valvular and hypertensive heart disease were not included in the DCM definition, irrespective of LV cavity size.

Relative wall thickness (RWT) was calculated in end-diastole by: (interventricular septal wall thickness [IVSd] + posterior wall thickness [PWd])/LVIDd. Left ventricular mass (LVM) was derived from the following equation: 0.8(1.04[LVIDd + PWd + IVSd]^3^ − [LVIDd]^3^) + 0.6. Remodelling measures were then indexed to body surface area (BSA). BSA was calculated using the Mosteller formula^79^: Body surface area (BSA) = square root of (height (cm) × weight (kg)/3600). Concentric remodelling was defined as RWT ≥ 0.42 and hypertrophy was defined as LVMi > 95 g/m^2^^75,76^. Left atrial dilatation was defined using the American and European 2015^76^ (using indexed measures, if available) and 2006 guidelines^80^ (when indexed measures were not available). This definition is summarised in Supplementary Table S11.

Echocardiography parameters were used from the most recent pre-pregnancy scan or, when this was not available, < 12 weeks’ gestation.

### Obstetric classifications

Pre-eclampsia was confirmed by documented diagnosis in the case notes or clinic letters. All cases met the International Society for the Study of Hypertension in Pregnancy (ISSHP) criteria for diagnosis^81^: new or worsening hypertension > 20 weeks and proteinuria or other suggestive features (abnormal haematological or biochemical parameters or FGR). Severe pre-eclampsia was defined as maximum blood pressure ≥ 160/110 mmHg, alanine aminotransferase > 100U/L, creatinine > 100 µmol/L or platelets < 100 × 10^9^/L. Data for birthweight centile customisation was not available for all women. Therefore, the World Health Organization (WHO) population Z score was used^82^. Small-for-gestational-age (SGA; birthweight < 10th centile) equated to a Z score < − 1.282 and FGR (birthweight < 3rd centile)^83^ equated to a Z score < − 1.881^82^.

Population pre-eclampsia prevalence of 4.6% was derived from Abalos et al.’s systematic review^45^. Preterm pre-eclampsia prevalence (requiring delivery < 37 weeks; 0.7%) was derived from the ASPRE trial^46^, in which 4.0% of the population were high-risk for pre-eclampsia, according to NICE^44^. Population rates of SGA (18.2%), FGR (9.5%), preterm delivery (< 37 weeks; 8.2%) and preterm FGR (< 37 weeks; 1.5%) were derived from 5-year data (2016–2020) from Saint Mary’s Hospital, Manchester, UK^84^.

### Outcomes

The primary outcome was to determine the prevalence of pre-eclampsia in women with pre-existing cardiac impairment, compared with the general population. Pre-specified secondary outcomes included: (1) the prevalence of FGR and SGA in women with pre-existing cardiac impairment, compared with the general population; the prevalence of pre-eclampsia, FGR and SGA depending on (2) primary cardiac diagnosis and (3) severity of LV impairment (by LVEF); (4) the relationship between gestation at birth/birthweight Z score and primary cardiac diagnosis/severity of LV impairment/other echocardiography parameters. An appropriate published core outcome set was not available and therefore not used in this study.

Cardiovascular endpoints included acute heart failure, pulmonary oedema, sustained arrhythmia, stroke, angina, myocardial infarction and cardiac arrest. Major adverse cardiovascular event (MACE) was defined by a composite outcome of stroke, myocardial infarction or maternal death.

### Statistical analysis

Statistical analyses were performed using Stata v.14.2. Baseline characteristics of the cohort were represented as mean ± standard deviation/median (range) as appropriate for continuous data, or counts (percentage) for categorical data. Prevalence of primary and secondary outcomes were compared against quoted population prevalence, as described in the literature^45,46^, using equality of proportions test. Prevalence of these outcomes was also compared between groups using Chi-square test. Univariate analysis was used to identify those factors significantly associated with pregnancy outcome. Heavily skewed variables were log-transformed prior to analysis. Multivariable regression analyses allowed adjustment for confounding factors. Analyses were performed for the whole cohort and repeated for the DCM subgroup, aiming to reduce heterogeneity (in particular, by removing the functional and haemodynamic consequences of structural heart disease).

The prevalence of pre-eclampsia in the general population is 4.6%^45^. In order to identify a twofold increase in pre-eclampsia in this cohort (≥ 9.2%) compared with the general population, a sample size of 245 women was required at 80% power, α 0.05. From the initial single centre cohort study at Saint Mary’s Hospital 66 eligible cases were identified. It was therefore anticipated that 12 additional sites would be needed (recruiting 15 per site) to reach the target sample size.

### Ethics approval

The protocol was approved by the UK HRA (without need for ethical committee review; IRAS ID 261380), the Australian Human Research Ethics Committees (HREC/60940/MonH-2020-203642) and the Research and Innovation teams at each site.

## Supplementary Information

Supplementary Information.

## Data Availability

The datasets generated during and/or analysed during the current study are available from the corresponding author on reasonable request.
