# Supplementary material for: Prevalence of pre-eclampsia and adverse pregnancy outcomes in women with pre-existing cardiomyopathy: a multi-centre retrospective cohort study
Source: Sci Rep. 2023 Jan 4;13:153. doi: 10.1038/s41598-022-26606-z (PMC9813256; doi:10.1038/s41598-022-26606-z)
Supplement: Supplementary file 1 — Supplementary Information. [file 41598_2022_26606_MOESM1_ESM.pdf]

Prevalence of pre-eclampsia and adverse pregnancy outcomes in women with pre-existing cardiomyopathy: a multi-centre retrospective cohort study.

Laura Ormesher[1,2], Sarah Vause[2], Suzanne Higson[3], Anna Roberts[2], Bernard Clarke[3,4], Stephanie Curtis[5], Victoria Ordonez[5], Faiza Ansari[6], Thomas R. Everett[6], Claire Hordern[7], Lucy Mackillop[7], Victoria Stern[8], Tessa Bonnett[8], Alice Reid[9], Suzanne Wallace[9], Ebruba Oyekan[10], Hannah Douglas[10], Matthew Cauldwell[11], Maya Reddy[12], Kirsten Palmer[12], Maggie Simpson[13], Janet Brennand[13,14], Laura Minns[15], Leisa Freeman[15], Sarah Murray[16], Nirmala Mary[16], James Castleman[17], Katie R. Morris[17,18], Elizabeth Haslett[19], Christopher Cassidy[19], Edward D Johnstone[1,2], Jenny E Myers[1,2].

1. Maternal & Fetal Health Research Centre, Division of Developmental Biology and Medicine, University of Manchester, UK.
2. Saint Mary's Hospital, Manchester University NHS Foundation Trust, UK.
3. Manchester Heart Centre, Manchester University NHS Foundation Trust, UK.
4. Division of Cardiovascular Sciences, University of Manchester, UK
5. Bristol Heart Institute, Bristol Royal Infirmary, Bristol, UK
6. Leeds Teaching Hospitals NHS Trust, Leeds, UK.
7. Oxford University Hospitals NHS Foundation Trust, Oxford, UK
8. Academic Unit of Developmental and Reproductive Medicine, University of Sheffield, United Kingdom.
9. Department of Obstetrics, Nottingham University Hospitals NHS Trust, UK.
10. Guy's and St Thomas' NHS Foundation Trust, London, UK
11. St George's University Hospitals NHS Foundation Trust, London, UK.
12. Monash Women's, Monash Health, Monash University, Australia.
13. Scottish Adult Congenital Cardiac Service, Golden Jubilee National Hospital, Glasgow, UK.
14. Queen Elizabeth University Hospital, Glasgow, UK
15. Department of Cardiology, Norfolk& Norwich University Hospital Foundation Trust, *Norwich*, UK.
16. Royal Infirmary of Edinburgh, NHS Lothian University Hospitals Division, Edinburgh, UK.
17. Birmingham Women's and Children's Hospital NHS Foundation Trust, Birmingham, UK.
18. Institute of Applied Health Research, University of Birmingham, Birmingham, UK.
19. Blackpool Teaching Hospitals NHS Foundation Trust, Blackpool, UK.

Supplementary Table S1: Recruitment from different sites.

City	Participating Hospital	Number of pregnancies included
**Birmingham**	Birmingham Women's Hospital	3
**Blackpool**	Blackpool teaching Hospitals NHS Foundation Trust	1
**Bristol**	Bristol Royal Infirmary	51 (all DCM)
**Edinburgh**	NHS Lothian	6
**Glasgow**	Queen Elizabeth University Hospital	10
**Leeds**	Leeds Teaching Hospitals NHS Trusts	35
**London**	St Thomas' Hospital	12
**Manchester**	St Mary's Hospital	99
**Melbourne**	Monash	11
**Norwich**	Norfolk and Norwich University Hospital NHS Foundation Trust	7
**Nottingham**	Nottingham University Hospitals NHS Trust	13
**Oxford**	John Radcliffe Hospital	19
**Sheffield**	Sheffield Teaching Hospitals NHS Foundation Trust	15

NHS, National Health Service; DCM, dilated cardiomyopathy.

Supplementary Table S2: Concomitant medication before and during pregnancy.

Echocardiography parameters	DCM (n=156)		All (n=282)	
	**Before pregnancy**	**During pregnancy**	**Before pregnancy**	**During pregnancy**
**ACE inhibitor / ARB**	74/143 (51.7%)		104/271 (38.4%)	
**ß blockers**	78/147 (53.1%)	85/152 (55.9%)	130/269 (48.3%)	134/277 (48.4%)
**Ca channel blockers**	4/151 (2.6%)	2/151 (1.3%)	9/277 (3.2%)	6/277 (2.2%)
**α blockers**	3/151 (2.0%)	4/151 (2.6%)	4/277 (1.4%)	5/277 (1.8%)
**Diuretics**	28/150 (18.7%)	34/149 (22.8%)	36/275 (13.1%)	45/275 (16.4%)
**Heparin**	14/148 (9.5%)	44/149 (29.5%)	20/274 (7.3%)	68/275 (24.7%)
**Vitamin K antagonists**	7/150 (4.7%)	2/153 (1.3%)	16/276 (5.8%)	3/279 (1.1%)
**Aspirin**	30/152 (19.7%)	58/134 (43.3%)	56/278 (20.1%)	102/257 (39.7%)

Frequencies: n/N (%)
Denominators vary between variables due to missing data.
ACE, angiotensin converting enzyme; ARB, angiotensin receptor blocker; Ca, calcium.

Supplementary Table S3: Baseline characteristics of the women who developed pre-eclampsia.

Cardiac diagnosis	LVEF impairment	Age (years)	BMI (kg/ m^2)	Booking BP (mmHg)	Pre-eclampsia risk factors	Antenatal aspirin	Obstetric history	Birthweight Z score	Gestation at delivery (completed weeks)
**Congenital heart disease (TGA)**	Borderline	28	37.2	120/78	2 moderate risk factors (nulliparous, ↑ BMI)	No	Nulliparous	-1.67	38
**Hypertensive cardiomyopathy**	Impaired	25	25.3	112/72	Hypertension, CKD	Yes	Nulliparous	-1.38	38
**Ischaemic heart disease**	Borderline	40	21.2	145/77	Previous PE, hypertension, CKD, age	Yes	Para 2; Previous PE, FGR	-1.07	33
**DCM (post-viral)**	Borderline	41	44.8	130/88	Age, hypertension	Yes	Para 1; no previous pre-eclampsia / FGR	-2.85	32
**DCM secondary to chemotherapy**	Borderline	19	23.7	90/67	Nulliparous	No	Nulliparous	-0.13	38
**DCM secondary to Libman-Sacks endocarditis linked to APS**	Severe	36	22.7	132/77	Previous pre-eclampsia, APS, CKD	Yes	Para 1; Previous pre-eclampsia	-1.93	33
**DCM secondary to sickle cell disease**	Borderline	33	17.9	114/70	CKD	Yes	Nulliparous	-2.93	35
**DCM; cardiac transplant for restrictive cardiomyopathy**	Borderline	29	24.1	106/70	CKD	No	Para 1; Previous FGR	-0.88	37
**VSD**	Impaired	28	46.6	130/81	Previous PE	Yes	Para 1; Previous PE	1..29	40
**VSD**	Impaired	28	30.9	104/64	Nil	Yes	Para 1	0.50	37
**Congenital heart disease (TOF)**	Impaired	28	21.8	130/84	Nulliparous	No	Nulliparous	1.90	37
**Coarctation of aorta**	Borderline	33	40.0	135/88	↑ BMI	Yes	Para 1	-0.64	38
**ASD**	Impaired	18	25.5	112/80	Nulliparous	No	Nulliparous	-1.69	35

Each row represents an individual woman.

LVEF, left ventricular ejection fraction; BMI, body mass index; BP, blood pressure; mmHg, millimetres of mercury; TGA, transposition of the great arteries;

CKD, chronic kidney disease; PE, pre-eclampsia; FGR, fetal growth restriction; DCM, dilated cardiomyopathy; APS, antiphospholipid syndrome; TOF, tetralogy of Fallot; ASD, atrial septal defect.

Supplementary Table S4: Prevalence of adverse pregnancy outcome compared with the background population.

Adverse pregnancy outcome	Background population	DCM	P value	Whole cohort	P value
**Pre-eclampsia**	4.6 (2.7 - 8.2)	3.2 (0.4 - 6.0)	0.40	4.6 (2.2 - 7.0)	0.99
**Preterm pre-eclampsia**	0.7 (0.6 - 0.8)	1.9 (0.0 - 4.1)	0.07	**1.8 (0.2 - 3.3)**	**0.03**
**Preterm delivery**	**8.2 (8.0 - 8.5)**	**28.3 (21.1 - 35.4)**	**<0.001**	**27.3 (22.1 - 32.6)**	**<0.001**
**SGA**	**18.2 (17.9 - 18.6)**	**29.2 (21.7 - 36.6)**	**0.001**	**32.0 (26.4 - 37.5)**	**<0.001**
**FGR**	**5.5 (5.3 - 5.7)**	**14.6 (8.8 - 20.3)**	**<0.001**	**15.2 (10.9 - 19.5)**	**<0.001**
**Preterm FGR**	**1.5 (1.4 - 1.6)**	**7.7 (3.7 - 11.7)**	**<0.001**	**7.4 (4.3 - 10.5)**	**<0.001**

Prevalence: % (95% Confidence interval).
P values represent comparison between whole cohort / DCM subgroup and the background population using equality of proportions test.
Bold text indicates statistical significance (p<0.05).
SGA, small-for-gestational-age (<10th centile_; FGR, fetal growth restriction (<3rd centile); preterm < 37 weeks' gestation.

Supplementary Table S5: Prevalence of adverse pregnancy outcome depending on severity of LVEF impairment.

Severity of LVEF impairment	Pre-eclampsia	SGA < 10th centile	FGR < 3rd centile	Delivery < 37 weeks	Delivery < 34 weeks
**DCM**					
**Borderline (n=63)**	4/64 (6.3%)	19/57 (33.3%)	9/57 (15.8%)	18/61 (29.5%)	4/61 (6.6%)
**Impaired (n=62)**	0/66 (0.0%)	13/62 (21.0%)	8/62 (12.9%)	15/65 (23.1%)	4/65 (6.2%)
**Severely impaired (n=26)**	1/26 (3.8%)	10/25 (40.0%)	4/25 (16.0%)	10/26 (38.5%)	4/26 (15.4%)
**P value**	0.13	0.14	0.88	0.33	0.30
**All**					
**Borderline (n=116)**	9/140 (6.4%)	41/132 (31.1%)	21/132 (15.9%)	36/147 (24.5%)	12/127 (8.8%)
**Impaired (n=99)**	3/110 (2.8%)	31/106 (29.2%)	14/106 (13.2%)	28/109 (25.7%)	12/109 (11.0%)
**Severely impaired (n=32)**	1/32 (3.1%)	14/31(45.2%)	6/31 (19.3%)	12/32 (37.5%)	6/32 (18.8%)
**P value**	0.35	0.24	0.67	0.39	0.26

Frequencies: n/N (%)
Denominators vary between variables due to missing data.
Severity of LVEF impairment was classified as: borderline (50-54%), impaired (36-49%) and severe (≤35%)[1].
P values represent comparison between LVEF impairment categories using Chi-square test.
LVEF, left ventricular ejection fraction; SGA, small for gestational age; FGR, fetal growth restriction; DCM, dilated cardiomyopathy.

Supplementary Table S6: Relationship between echocardiography parameters and birthweight Z score.

Echocardiography parameters	DCM (n=156)			All (n=282)		
	N	Coefficient (95% C.I.)	P value	N	Coefficient (95% C.I.)	P value
**LVM (increment 10g)**	80	0.00 (-0.01 - 0.00)	0.68	152	0.00 (0.00 -0.00)	0.95
**LVMi (increment 5g/m²)**	76	-0.01 (-0.02 - 0.00)	0.16	122	0.00 (-0.01 - 0.01)	0.45
**RWT (increment 0.1)**	81	-0.57 (-4.08 - 2.95)	0.75	159	-0.87 (-3.00 - 1.26)	0.42
**E/A (increment 0.2)**	52	-0.32 (-0.81 - 0.16)	0.18	92	-0.02 (-0.23 - 0.18)	0.83
**E/E' (increment 1)**	28	-0.08 (-0.22 - 0.67)	0.28	48	-0.02 (-0.09 - 0.06)	0.67
**Concentric hypertrophy* (n=6)**	76	-1.04 (-2.50 - 0.42)	0.16	122	-0.68 (-1.67 - 0.32)	0.18
**Concentric remodelling* (n=8)**		0.62 (-1.14 - 2.39)	0.48		0.27 (-0.66 - 1.19)	0.57
**Eccentric hypertrophy* (n=49)**		0.15 (-0.43 - 0.73)	0.60		0.10 (-0.35 - 0.54)	0.67
**Mild LA enlargement† (n=11)**	39	0.02 (-0.04 - 0.07)	0.54	66	-0.07 (-0.89 - 0.76)	0.88
**Moderate LA enlargement† (n=4)**		0.03 (-0.06 - 0.11)	0.52		-0.15 (-1.43 - 1.14)	0.82
**Severe LA enlargement† (n=1)**		-0.09 (-0.24 - 0.05)	0.19		-1.73 (-4.23 - 0.78)	0.17
**Aortic stenosis‡ (n=16)**	-	-	-	219	0.42 (-0.17 - 1.01)	0.17
**Aortic regurgitation‡ (n=37)**	-	-	-	219	0.28 (-0.12 - 0.68)	0.17
**Mitral stenosis‡ (n=7)**	-	-	-	221	-0.49 (-1.26 - 0.28)	0.21
**Mitral regurgitation‡ (n=79)**	-	-	-	216	-.30 (-0.07 - 0.67)	0.11
**Pulmonary stenosis‡ (n=4)**	-	-	-	220	0.19 (-0.95 - 1.34)	0.74
**Pulmonary regurgitation‡ (n=27)**	-	-	-	214	0.04 (-0.41 - 0.49)	0.86
**Tricuspid stenosis‡ (n=0)**	-	-	-	219	-	-
**Tricuspid regurgitation‡ (n=51)**	-	-	-	211	-0.13 (-0.48 - 0.22)	0.48
**TAPSE (increment 1mm)**	37	0.01 (-0.17 - 0.18)	0.95	92	**0.04 (0.00 - 0.08)**	**0.04**

*Compared to normal LV size.
†Compared to normal LA size.
‡Compared to none / physiological valvular regurgitation / stenosis.
N describes the number of observations included in the analysis.
n describes the number of pregnancies affected by the condition.

DCM, dilated cardiomyopathy; C.I., confidence interval; LVM, left ventricular mass; LVMi, LVM indexed to body surface area; RWT, relative wall thickness; E/A, early to late diastolic filling ratio; E/E’, early diastolic filling to early diastolic mitral annular velocity ratio; LA, left atrium; TAPSE, tricuspid annular plane systolic excursion; LV, left ventricular.

Supplementary Table S7: Relationship between echocardiography parameters and gestation at delivery (log-transformed).

Echocardiography parameters	DCM (n=156)			All (n=282)		
	N	Coefficient (95% C.I.)	P value	N	Coefficient (95% C.I.)	P value
**LVM (increment 10g)**	82	0.00 (0.00 - 0.00)	0.95	155	0.00 (0.00 - 0.00)	0.01
**LVMi (increment 5g/m²)**	78	0.00 (0.00 - 0.00)	0.53	125	0.00 (0.00 - 0.00)	0.34
**RWT (increment 0.1)**	83	0.05 (-0.12 - 0.23)	0.56	162	-0.14 (-0.30 - 0.02)	0.09
**E/A (increment 0.2)**	54	0.00 (-0.02 - 0.02)	0.78	95	0.00 (-0.02 - 0.02)	0.93
**E/E' (increment 1)**	30	0.00 (-0.01 - 0.00)	0.56	50	5.57 (5.50 - 5.63)	0.79
**Concentric hypertrophy* (n=6)**	78	-0.02 (-0.10 - 0.05)	0.57	125	**-0.10 (-0.18 - -0.03)**	**0.009**
**Concentric remodelling* (n=8)**		0.02 (0.07 - 0.11)	0.69		-0.02 (-0.09 - 0.05)	0.65
**Eccentric hypertrophy* (n=49)**		0.00 (-0.03 - 0.03)	0.89		-0.02 (-0.05 - 0.02)	0.32
**Mild LA enlargement† (n=11)**	39	0.02 (-0.04 - 0.07)	0.54	66	0.03 (-0.03 - 0.09)	0.31
**Moderate LA enlargement† (n=4)**		0.03 (-0.06 - 0.11)	0.52		0.04 (-0.05 - 0.13)	0.33
**Severe LA enlargement† (n=1)**		-0.09 (-0.24 - 0.05)	0.19		-0.08 (-0.25 - 0.09)	0.36
**Aortic stenosis (n=16)**	-	-	-	228	0.02 (-0.02 - 0.07)	0.28
**Aortic regurgitation‡ (n=37)**	-	-	-	228	0.02 (-0.01 - 0.05)	0.25
**Mitral stenosis‡ (n=7)**	-	-	-	230	-0.04 (-0.10 - 0.01)	0.14
**Mitral regurgitation‡ (n=79)**	-	-	-	225	0.01 (-0.01 - 0.04)	0.32
**Pulmonary stenosis‡ (n=4)**	-	-	-	229	0.02 (-0.07 - 0.11)	0.71
**Pulmonary regurgitation‡ (n=27)**		-	-	223	0.01 (-0.03 - 0.04)	0.69
**Tricuspid stenosis‡ (n=0)**	-	-	-	228	-	
**Tricuspid regurgitation‡ (n=51)**	-	-	-	220	0.00 (-0.03 - 0.02)	0.93
**TAPSE (increment 1mm)**	40	0.00 (-0.01 - 0.01)	0.88		0.00 (0.00 - 0.00)	0.20

*Compared to normal LV size.
†Compared to normal LA size.
‡Compared to none / physiological valvular regurgitation / stenosis.
Gestation at delivery was skewed and therefore log-transformed for the purpose of these analyses.
N describes the number of observations included in the analysis.
n describes the number of pregnancies affected by the condition.
Bold text indicates statistical significance (p<0.05).

DCM, dilated cardiomyopathy; C.I., confidence interval; LVM, left ventricular mass; LVMi, LVM indexed to body surface area; RWT, relative wall thickness; E/A, early to late diastolic filling ratio; E/E', early diastolic filling to early diastolic mitral annular velocity ratio; LA, left atrium; TAPSE, tricuspid annular plane systolic excursion; LV, left ventricular.

Supplementary Table S8: Relationship between valvular dysfunction and pre-eclampsia prevalence, after adjustment for booking MAP and pre-eclampsia risk factors.

**Echocardiography parameters**	**DCM**			**All**		
	**N**	**Adjusted OR (95% C.I.)**	**P value**	**N**	**Adjusted OR (95% C.I.)**	**P value**
**Aortic stenosis (n=16)**	82	-	-	170	3.90 (0.84 - 18.22)	0.08
**Mitral stenosis (n=7)**	**83**	**57.72 (2.78 - 1197.47)**	**0.01**	172	3.70 (0.57 - 24.08)	0.17
**Pulmonary regurgitation (n=27)**	79	4.31 (0.48 - 38.73)	0.19	166	3.31 (0.83 - 13.23)	0.09

Compared to none / physiological valvular regurgitation / stenosis.
N describes the number of observations included in the analysis.
n describes the number of pregnancies affected by the condition.
Bold text indicates statistical significance ($p<0.05$).
MAP, mean arterial pressure; DCM, dilated cardiomyopathy; OR, odds ratio; C.I., confidence interval.

Supplementary Table S9: Maternal cardiac phenotype and fetal growth restriction risk factors, depending on antenatal ß blocker exposure.

Maternal characteristics	Exposed to ß blockers antenatally	Not exposed to ß blockers antenatally	P value
**Severity of LVEF impairment**			
**Borderline**	49/134 (36.6%)	88/143 (61.5%)	<0.001
**Impaired**	64/134 (47.8%)	44/143 (30.8%)	
**Severely impaired**	21/134 (15.7%)	11/143 (7.7%)	
**Primary cardiac diagnosis**			
**DCM**	87/134 (64.9%)	67/143 (46.9%)	<0.001
**Congenital heart disease**	5/134 (3.7%)	30/143 (21.0%)	
**Ischaemic heart disease**	10/134 (7.5%)	2/143 (1.4%)	
**Hypertensive cardiomyopathy**	2/134 (1.5%)	1/143 (0.7%)	
**Valvular disease**	13/134 (9.7%)	18/143 (12.6%)	
**Genetic heart disease without DCM**	12/134 (9.0%)	9/143 (6.3%)	
**Other acquired heart disease without DCM**	5/134 (3.7%)	16/143 (11.2%)	
**Risk factors for FGR**			
**Hypertension**	15/133 (11.3%)	5/141 (3.5%)	0.01
**Smoked during pregnancy**	29/126 (23.0%)	24/132 (18.2%)	0.34

Frequencies: n/N (%)
Denominators vary between variables due to missing data.
Severity of LVEF impairment was classified as: borderline (50-54%), impaired (36-49%) and severe (≤35%)[56].
P values represent comparison between pregnancies exposed to ß blockers antenatally and those not, using Chi-square test.
LVEF, left ventricular ejection fraction; DCM, dilated cardiomyopathy; FGR, fetal growth restriction.

Supplementary Table S10: Requested variables to be recorded from each case.

**Baseline maternal characteristics**	Age at delivery<br>Ethnicity<br>Booking height<br>Booking weight<br>Booking blood pressure<br>Smoking status at booking<br>Medical history (hypertension, renal disease, autoimmune disease, pre-existing proteinuria, pre-existing diabetes)<br>Primary cardiac diagnosis<br>NYHA functional status<br>Presence and cause of dilated cardiomyopathy<br>BNP / NTproBNP (within 1 year of conception)<br>Medication before and during pregnancy (ACE inhibitors, ARB, ß blockers, calcium channel blockers, α blockers, diuretics, heparin, vitamin K antagonists, aspirin)
**Obstetric history**	Gravidity<br>Parity<br>Previous pregnancy complications (including preterm birth, SGA, FGR and pre-eclampsia)
**Echocardiography parameters**<br><br>**(pre-pregnancy [most recent] and early pregnancy [<12 weeks' gestation])**	LVEF (discrete number / range / description)<br>Presence of valvular disease: which valve, quantified if present<br>Presence of HOCM<br>Cardiac remodelling: presence of eccentric hypertrophy / concentric remodelling / concentric hypertrophy<br>TAPSE<br>LVIDd<br>LVIDs<br>PWd<br>IVSd<br>E/A<br>E/E'<br>LAV<br>CO<br>SV
**Pregnancy outcome**	Gestational age at delivery<br>Birthweight<br>Infant sex<br>Mode of delivery<br>Indication for delivery<br>Outcome – livebirth / stillbirth / neonatal or infant death<br>Neonatal admission to NICU (yes / no; number of days, if available)<br>Pre-eclampsia/eclampsia/PIH (gestation at diagnosis; severity)<br>GDM<br>Placental abruption<br>PPROM<br>EBL
**Cardiac outcome**	Acute / worsening heart failure<br>Pulmonary oedema<br>Sustained arrhythmia<br>Stroke / angina / myocardial infarction / cardiac arrest

NYHA, New York Heart Association; BNP, brain natriuretic peptide; NTproBNP, N-terminal-pro hormone BNP; ACE, angiotensin converting enzyme; ARB, angiotensin II receptor blockers; SGA,

small for gestational age; FGR, fetal growth restriction; LVEF, left ventricular ejection fraction; HOCM, hypertrophic obstructive cardiomyopathy; LVIDd, left ventricular internal diameter end-diastole; LVIDs, left ventricular internal diameter end-systole; PWd, posterior wall thickness end-diastole; IVSd, interventricular septum thickness end-diastole; E/A, early to late diastolic filling ratio (mitral inflow Doppler indices); E/E', early diastolic filling to early diastolic mitral annular velocity ratio (mitral inflow and annular Doppler indices); LAV, left atrial volume; CO, cardiac output; SV, stroke volume; NICU, neonatal intensive care unit; PIH, pregnancy induced hypertension; GDM, gestational diabetes; PPROM, premature prolonged rupture of membranes; EBL, estimated blood loss.

Supplementary Table S11: Classification of left atrial dilatation.

**Classification**	**Left atrial diameter (cm)***	**Left atrial volume (mL)***	**Left atrial volume index (mL/m²)**
**Normal**	< 3.9	22 - 52	16 - 34
**Mild**	3.9 - 4.2	53 - 62	35 - 41
**Moderate**	4.3 - 4.6	63 - 72	42 - 48
**Severe**	≥ 4.7	≥ 73	> 48

*Derived from American and European 2006 recommendations[2].
†Derived from American and European 2015 recommendations[3].

References

1. Harkness A, Ring L, Augustine DX, Oxborough D, Robinson S, Sharma V. Normal reference intervals for cardiac dimensions and function for use in echocardiographic practice: a guideline from the British Society of Echocardiography. *Echo Res Pract*. 2020;7(1):G1-G18. doi:10.1530/ERP-19-0050

2. Lang RM, Bierig M, Devereux RB, et al. Recommendations for chamber quantification. *Eur J Echocardiogr*. 2006;7(2):79-108. doi:10.1016/j.euje.2005.12.014

3. Lang RM, Badano LP, Victor MA, et al. Recommendations for cardiac chamber quantification by echocardiography in adults: An update from the American Society of Echocardiography and the European Association of Cardiovascular Imaging. *J Am Soc Echocardiogr*. 2015;28(1):1-39.e14. doi:10.1016/j.echo.2014.10.003
